# Protective Effect of Dinitrosyl Iron Complexes with Glutathione in Red Blood Cell Lysis Induced by Hypochlorous Acid

**DOI:** 10.1155/2019/2798154

**Published:** 2019-04-08

**Authors:** Konstantin B. Shumaev, Irina V. Gorudko, Olga V. Kosmachevskaya, Daria V. Grigorieva, Оleg M. Panasenko, Anatoly F. Vanin, Alexey F. Topunov, Maria S. Terekhova, Alexey V. Sokolov, Sergey N. Cherenkevich, Enno K. Ruuge

**Affiliations:** ^1^Research Center of Biotechnology of the Russian Academy of Sciences, Bach Institute of Biochemistry, Moscow 119071, Russia; ^2^National Medical Research Centre for Cardiology, Moscow 121552, Russia; ^3^Belarusian State University, Minsk 220030, Belarus; ^4^Federal Research and Clinical Center of Physical-Chemical Medicine of Federal Medical Biological Agency, Moscow 119435, Russia; ^5^Pirogov Russian National Research Medical University, Moscow 117997, Russia; ^6^Russian Academy of Sciences, Semenov Institute of Chemical Physics, Moscow 119991, Russia; ^7^Institute of Experimental Medicine, Saint Petersburg 197376, Russia; ^8^Lomonosov Moscow State University, Faculty of Physics, Moscow 119234, Russia

## Abstract

Hypochlorous acid (HOCl), one of the major precursors of free radicals in body cells and tissues, is endowed with strong prooxidant activity. In living systems, dinitrosyl iron complexes (DNIC) with glutathione ligands play the role of nitric oxide donors and possess a broad range of biological activities. At micromolar concentrations, DNIC effectively inhibit HOCl-induced lysis of red blood cells (RBCs) and manifest an ability to scavenge alkoxyl and alkylperoxyl radicals generated in the reaction of HOCl with *tert*-butyl hydroperoxide. DNIC proved to be more effective cytoprotective agents and organic free radical scavengers in comparison with reduced glutathione (GSH). At the same time, the kinetics of HOCl-induced oxidation of glutathione ligands in DNIC is slower than in the case of GSH. HOCl-induced oxidative conversions of thiolate ligands cause modification of DNIC, which manifests itself in inclusion of other ligands. It is suggested that the strong inhibiting effect of DNIC with glutathione on HOCl-induced lysis of RBCs is determined by their antioxidant and regulatory properties.

## 1. Introduction

Hypochlorous acid (HOCl) (its salt is generally referred to as hypochlorite (OCl^−^)) is one of the main molecular precursors of free radicals in living organisms [1, 2]. In the human organism, HOCl is formed in the reaction of H_2_O_2_ with chloride (Cl^−^) catalyzed by myeloperoxidase (MPO) from phagocytic cells, viz., neutrophils and monocytes [2, 3]. HOCl, being a powerful oxidant, plays a key role in the elimination of pathogenic microorganisms. By virtue of its high reactivity, HOCl comes into contact with many biologically important molecules and thus exerts cytotoxic effects by provoking the development of many severe conditions associated with inflammation [2, 4]. Human red blood cells (RBCs) are widely used as a model for studying general mechanisms of cell injury under conditions of phagocyte-induced oxidative stress [3, 5–7]. The interaction of hypohalous acids (HOCl, HOBr) with RBСs yields protein-bound free radical species, which change the plasma membrane and, as a consequence, induce deformation and lysis of RBCs [1, 5, 7]. On the other hand, HOCl-induced oxidative damage to red blood cells is stimulated by such prooxidants as nitrite or *tert*-butyl hydroperoxide [7].

**Figure 1:**
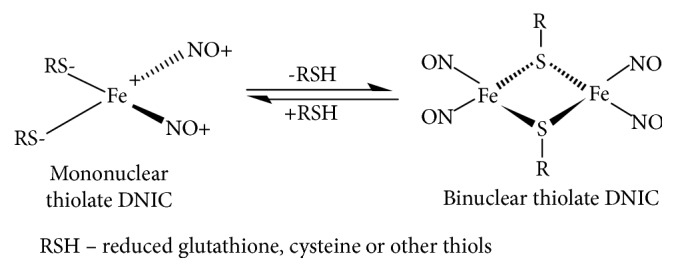
The equilibrium between mononuclear and binuclear dinitrosyl iron complexes (DNIC) with thiolate ligands.

Recent studies demonstrated that RBCs strongly influence the metabolic characteristics of nitric oxide (NO), which possesses unique regulatory capabilities [8–11]. Damage of RBCs under conditions of oxidative stress leads to hemolysis [12–16]; this and the formation of hemoglobin-rich microparticles are accompanied by enhanced generation of reactive oxygen species (ROS) and disturbances in the signaling function of NO [10, 11, 17]. A crucial role in stabilization and transport of NO to circulating blood is played by dinitrosyl iron complexes (DNIC) [18–20]. The latter contain [Fe(NO)_2_] fragments able to bind to low-molecular thiols (e.g., glutathione and cysteine) or amino acid residues of proteins. Under physiological conditions, mononuclear thiol-containing DNIC are at the state of equilibrium with binuclear DNIC represented by appropriate Roussin's red salt thioethers (Figure 1) [20–22]. Earlier, it was found that DNIC are formed in the course of NO generation by activated macrophages [23–26]. DNIC with glutathione possess a number of unique characteristics, i.e., they exert hypotensive and vasodilator effects, reduce the size of the infarction zone in isolated hearts, inhibit platelet aggregation, accelerate skin wound healing, and suppress endometriosis and apoptosis in cultured animal cells [18, 20]. Our previous studies established that DNIC increase the elasticity of RBCs and inhibit their detergent-induced lysis [27]. Moreover, DNIC fulfil the function of NO and nitrosonium ion (NO^+^) donors; this remarkable capability determines their high physiological activity [20, 22, 28]. The antioxidant and cytoprotective effects of DNIC on living cells and systems under conditions of oxidative stress [28–33] prompt a conclusion that in-depth study of DNIC effects on HOCl-induced lysis of RBСs is a task of paramount importance.

## 2. Materials and Methods

### 2.1. Reagents

Sodium hypochlorite (NaOCl), *tert*-butyl hydroperoxide solution, and reduced glutathione were from Sigma-Aldrich (St. Louis, MO). ThioGlo 1 (10-(2,5-dihydro-2,5-dioxo-1H-pyrrol-1-yl)-9-methoxy-3-oxo-3H-naphthol[2,1-b]pyran-2-carboxylic acid methyl ester) and DEPMPO (5-diethoxy-phosphoryl-5-methyl-1-pyrroline N-oxide) were from Calbiochem (San Diego, CA). Other reagents were from Sigma-Aldrich (USA).

The concentration of commercial NaOCl solutions was determined as OClˉ concentration measured spectrophotometrically at pH 12.0, taking the molar extinction coefficient (*ε*_292_) equal to 350 M^−1^ cm^−1^ [34]. Assuming that *р*K*_α_* for HOCl is ~7.5 [34] and that at physiological pH about 50% of HOCl exists in the protonated form, while the resting 50% is in the dissociated form, hereinafter under the term “HOCl” is understood the HOCl/OClˉ mixture present in the test solution. The working solution of HOCl was prepared immediately before assay by dissolution of the commercial preparation in 10 mM Na-phosphate buffer pH 7.4 containing 140 mM NaCl.

Native myeloperoxidase (MPO) was isolated from extracts of frozen leukocytes of healthy donors as described elsewhere [35]. The purity of MPO preparations was estimated by the Reinheit Zahl (RZ) value (the 430/280 nm absorbance ratio ~0.85).

### 2.2. Synthesis of Dinitrosyl Iron Complexes

DNIC with phosphate ligands were synthesized in a Thunberg tube by passing gaseous NO through a mixture containing FeSO_4_ and 100 mM Na,K-phosphate buffer (pH 6.8) as a ligand source [29, 30]. DNIC with thiol ligands were obtained by adding reduced glutathione (GSH) or cysteine to less stable DNIC with phosphate at the molar ratio of 2 : 1. Protein-bound DNIC were obtained by mixing cysteine- or glutathione-ligated DNIC with bovine serum albumin (BSA). The solutions of various DNIC were frozen in liquid nitrogen and stored until use.

### 2.3. Isolation of Human Red Blood Cells

Human red blood cells (RBCs) were isolated from donor blood stabilized with sodium citrate and washed by twofold centrifugation at 350 g in PBS containing 10 mM Na_2_HPO_4_/KH_2_PO_4_, 137 mM NaCl, and 2.7 mM KCl (pH 7.4).

### 2.4. Detection of RBC Lysis

The kinetics of RBC lysis was followed with the help of a PB 2201 spectrophotometer (SOLAR, Minsk, Belarus) by monitoring the changes in the optical density of the cell suspensions (~4 × 10^7^ cells/ml) at 670 nm. The concentration of RBC was chosen so that the initial optical density of the cell suspension was about 0.6 as the most optimal for measurement. To this end, 1 ml of washed RBC suspension was added to the measuring cuvette and thermostatted for 3-4 min at 37°C upon continuous stirring in the absence or in the presence of DNIC. Lysis of RBCs was initiated using two approaches. The first of them consisted in addition of 0.1–1 mM HOCl to RBC suspensions in PBS containing 1 mM СаСl_2_ and 0.5 mM MgCl_2_ (NB: the optical density at 670 nm did not exceed 0.7). In the second approach, RBC lysis was initiated by adding Н_2_О_2_ to RBC suspensions after their 2 min preincubation with MPO (50 nM) in 155 mM NaCl.

The rate of induced lysis of RBCs was determined from the rate of lysis calculated from the slope of the linear segment of the kinetic curve reflecting the decrease in the optical density at 670 nm.

### 2.5. EPR Assay

EPR spectra were measured at ambient temperature (25°C) using an X-band EPR spectrometer E-109E (Varian, USA). The instrument settings were as follows: modulation frequency, 100 kHz; time constant, 0.032; microwave power, 10 mW; microwave frequency, 9.15 GHz; and modulation amplitudes, 0.1 or 0.2 mT for DNIC and 0.1 mT for DEPMPO spin adducts.

### 2.6. Measurement of the Thiol Group with ThioGlo 1

Thiol groups were detected with the help of the fluorescent probe ThioGlo 1 [36, 37]. The fluorescence spectra of the ThioGlo 1 adduct with glutathione were recorded on a RF-5301 PC spectrofluorimeter (Shimadzu, Japan). The excitation and emission of the adduct were 379 and 506 nm, respectively. The stock solution of ThioGlo 1 in anhydrous DMSO (2 mM) was stored in the dark at -20°С. Prior to assay, 5 *μ*l of a ThioGlo 1 solution in DMSO was added to a 5 *μ*l of a mixture containing GSH or DNIC with glutathione and incubated for 3 min at ambient temperature. Fluorescence spectra were measured after the addition of 490 *μ*l of 10 mM Na,K-phosphate buffer (pH 7.4) to the test solution.

### 2.7. Statistical Analysis

The experimental results were expressed as mean ± SEM (M ± m) from 4 to 6 independent measurements. Statistical analysis was performed using Origin 7 and Origin 8 software packages (OriginLab Corporation, USA). The value of *P* < 0.05 was taken as statistically significant.

## 3. Results

### 3.1. Effect of DNIC with Glutathione on HOCl-Induced Lysis of RBCs

**Figure 2:**
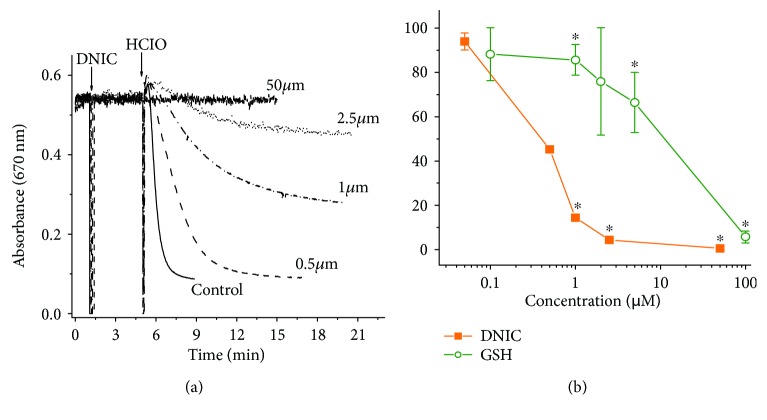
The effects of glutathione DNIC on HOCl-induced lysis of RBCs. Characteristic kinetic curves of hemolysis initiated by the addition of 0.25 mM HOCl in the absence and in the presence of different concentrations of DNIC (a). Dependence of the rate of RBC lysis on the presence of DNIC (orange) and GSH (green) in the incubation medium (b). The parameters of the HOCl-induced lysis were taken for 100%. ^∗^*P* < 0.05.

The lysis of RBCs under conditions of oxidative/halogenative stress was induced either by treatment of RBC suspensions with HOCl (the synthesis of the latter is catalyzed by myeloperoxidase (MPO)) or by adding MPO to RBC suspensions in the presence of its specific substrates (H_2_O_2_ and chloride). The characteristic kinetic curves for HOCl-induced lysis of RBCs in the absence and in the presence of different concentrations of DNIC are shown in Figure 2(a).

As can be seen, after the treatment of RBCs with DNIC, their resistance to HOCl increased considerably, while the rate of HOCl-induced lysis decreased with the increase in the DNIC concentration resulting in complete inhibition of RBC lysis at 50 *μ*M DNIC (Figure 2(a)). Earlier, it was established that reduced glutathione (GSH) is one of the most effective antioxidants for RBCs [11, 15] by virtue of its high ability to scavenge HOCl; this reaction gives glutathione sulfonamide as the main product [2, 4, 38]. Considering that in our study DNIC contained two glutathione molecules as ligands, we set ourselves a task to examine whether or not the protective effect of DNIC is related to the ability of glutathione to interact with HOCl. The effect of GSH on RBC lysis in the presence of HOCl is shown in Figure 2(b). As can be seen, a significant (*P* < 0.05) increase in the resistance of RBCs to lysis was observed only in the presence of glutathione used at concentrations above 5 *μ*M (Figure 2(b)). At 2.5 *μ*M, DNIC with glutathione (which corresponded to 5 *μ*M GSH) decreased the rate of HOCl-induced lysis of RBCs more than 20-fold.

**Figure 3:**
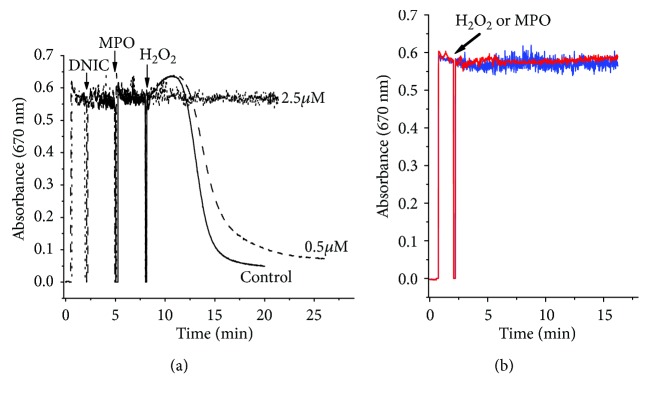
Effect of DNIC on hemolysis in HOCl production by myeloperoxidase (MPO). The characteristic kinetic curves of hemolysis induced by the addition of 50 nM MPO and 100 *μ*M Н_2_О_2_ to RBC suspensions in the absence and in the presence of 0.5 and 2.5 *μ*M DNIC with glutathione (a). In the presence of only MPO (blue) or hydrogen peroxide (red), the lysis of red blood cells does not occur (b).

The kinetics of RBC lysis induced by MPO, hydrogen peroxide, and Cl^−^ is shown in Figure 3. These data suggest that MPO catalyzing the oxidation of Cl^−^ by H_2_O_2_ to HOCl provoked RBC lysis (Figures 3(a) and 3(b)). The addition of 0.5 *μ*M DNIC to the incubation medium inhibited this process, while 2.5 *μ*M DNIC prevented it virtually completely (Figure 3(a)). These data unequivocally indicate that under conditions of simulated oxidative/halogenative stress DNIC with glutathione exert pronounced cytoprotective effect.

### 3.2. Antiradical Effect of DNIC with Glutathione

One of the most common reasons for RBC damage is the activation of free radical lipid peroxidation by hypohalous acids. Previous studies established that the reaction of HOCl with organic hydroperoxides (*tert*-butyl hydroperoxide, linoleic acid hydroperoxide) gives alkylperoxyl and alkoxyl radicals [2, 39–42]. These free radicals are generated in the presence of *tert*-butyl hydroperoxide in the following reactions [39, 41]:

(1) $$\begin{array}{c} {\left(С{Н}_{3}\right)}_{3}СОО\text{H}+\text{HOCl}⟶{\left(С{Н}_{3}\right)}_{3}С{ОО}^{\cdot }+{\text{Cl}}^{\cdot }+{\text{H}}_{2}\text{O}, \end{array}$$

(2) $$\begin{array}{c} 2{\left(С{Н}_{3}\right)}_{3}С{ОО}^{\cdot }⟶{\left(С{Н}_{3}\right)}_{3}\text{COOOOC}{\left({\text{CH}}_{3}\right)}_{3}⟶2{\left(С{Н}_{3}\right)}_{3}С{О}^{\cdot }\;{+}^{3}{\text{O}}_{2}. \end{array}$$

Interactions of fatty acid hydroperoxides with hypochlorite can also occur via following reactions [42]:

(3) $$\begin{array}{c} \text{ROOH}+{\text{OCl}}^{-}⟶\text{ROOCl}+{\text{H}}_{2}\text{O}, \end{array}$$

(4) $$\begin{array}{c} \text{ROOCl}⟶{\text{RO}}^{\cdot }{+}^{\cdot }\text{OCl}, \end{array}$$

(5) $$\begin{array}{c} \text{ROOCl}⟶{\text{ROO}}^{\cdot }{+}^{\cdot }\text{Cl}. \end{array}$$

In this case, alkoxyl (RO^·^) and alkylperoxyl radicals (ROO^·^) are produced due to the decomposition of chlorinated peroxide intermediates (ROOCl). In addition, ROO^·^ can be produced during the oxidation of organic hydroperoxides by the radical ^·^Cl.

Earlier, it was found that DNIC with different ligands are effective scavengers of free radicals generated in the course of decomposition of *tert*-butyl hydroperoxide [30, 43]. DNIC with glutathione also inhibited free radical oxidation of *β*-carotene induced by arachidonic acid hydroperoxide [44].

**Figure 4:**
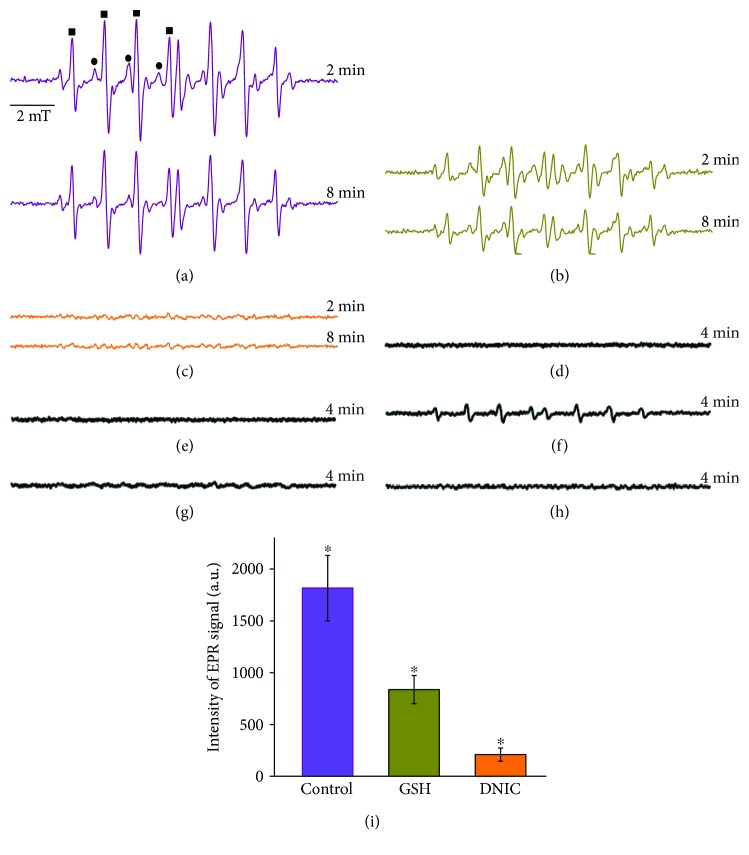
The effects of DNIC with glutathione and GSH on the magnitude of free radicals generated in the reaction between HOCl and *tert*-butyl hydroperoxide (t-BOOH). The EPR spectra were recorded in a reaction medium containing 7.2 mM t-BOOH, 0.75 mM HOCl, and 40 mM DEPMPO (a). The EPR spectra were recorded in the same medium as in (a) in the presence of 0.4 mM GSH (b) or 0.2 mM DNIC (c). The spectra represented a superposition of the EPR signals of spin trap adducts with free radicals (t-BOOH derivatives) including alkoxyl (■) and alkylperoxyl (●) radicals. The EPR spectra were recorded in the same medium as in (a), except that the latter contained 0.4 mM GSH (d) or 0.2 mM DNIC (e), but no t-BOOH. The spectrum (f) was recorded in a medium containing 40 mM DEPMPO, 0.75 mM HOCl, and 0.2 mM FeSO_4_, the spectrum (g)—in the same medium but without FeSO_4_, the spectrum (h)—in presence of only DEPMPO. The signals of the spin adducts were identified using the data from [45]. (i) The intensity of the signals of DEPMPO spin adducts obtained under conditions corresponding to the spectra (a–c) after 8-minute incubation. Signal intensity of the spin adducts was expressed as mean ± S.E. from three separate experiments. ^∗^*P* < 0.05.

In our study, adducts were formed as a result of interaction of the spin trap DEPMPO with free radical intermediates of the reaction between *tert*-butyl hydroperoxide and HOCl (Figure 4(a)). However, the formation of DEPMPO spin adducts faded after the addition of DNIC to the reaction medium (Figure 4(c)). Under these conditions, the antiradical effect of DNIC with glutathione was much more pronounced than in the case of free GSH (Figures 4(b), 4(c), and 4(i)).

This effect can be attributed to the interaction of alkylperoxyl and alkoxyl radicals with nitric oxide generated from DNIC:

(6) $$\begin{array}{c} {\text{ROO}}^{\cdot }+{\text{NO}}^{\cdot }⟶\text{ROONO}⟶{\text{RO}}^{\cdot }+{{\text{NO}}_{2}}^{\cdot } \end{array}$$

(7) $$\begin{array}{c} {\text{RO}}^{\cdot }+{\text{NO}}^{\cdot }⟶{\text{RNO}}_{2} \end{array}$$

(8) $$\begin{array}{c} {\text{RO}}^{\cdot }+{{\text{NO}}_{2}}^{\cdot }⟶{\text{RONO}}_{2}. \end{array}$$

Previous studies demonstrated that these diffusion-controlled reactions (*k* = 1 − 3 × 10^9^ M^−1^ s^−1^) culminate in complete inhibition of chain reactions responsible for free radical oxidation [46–48]. Meanwhile, the free radicals generated in reactions (1)–(5) scavenged effectively glutathione ligands in DNIC. With this in mind, we undertook to examine the feasibility of formation of thiyl radicals (RS^·^) during GSH oxidation by peroxynitrite [49] or products of myoglobin interaction with *tert*-butyl hydroperoxide using DEPMPO as a spin trap [43]. These studies established that in this series of our experiments thiyl-DEPMPO adducts could hardly be formed after the addition of HOCl to GSH or DNIC with glutathione (Figures 4(d) and 4(e)).

### 3.3. Effects of HOCl on Glutathione-Containing and Albumin-Bound DNIC

**Figure 5:**
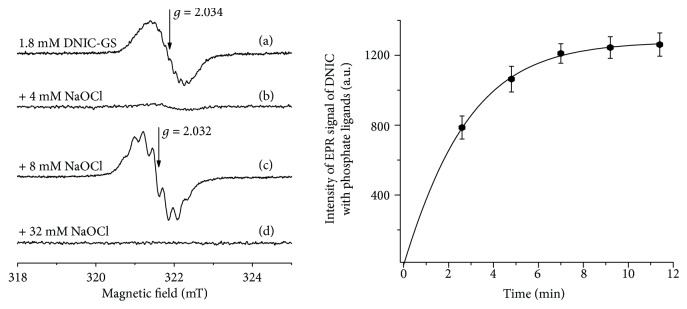
Interaction between hypochlorous acid and DNIC with glutathione ligands (DNIC-GS). The EPR signal recorded in a reaction medium containing 100 mM Na,K-phosphate buffer (pH 7.4) and 1.8 mM DNIC with glutathione ligands (a). The ratio of iron and glutathione under these conditions was approximately 1 : 2.2. The EPR spectra were recorded 2.5 min after the addition of different concentrations of HOCl (b–d). The kinetics of formation of DNIC with phosphate ligands was recorded in a reaction medium containing 1.8 mM DNIC-GS and 8 mM HOCl (e).

Mononuclear DNIC with glutathione ligands represent paramagnetic complexes with a characteristic EPR signal at *g* = 2.034 (Figure 5(a)). This EPR signal was reduced after the addition of HOCl to DNIC with glutathione eventually resulting in its complete disappearance (Figure 5(b)). This effect can be attributed to the conversion of mononuclear (EPR-active) DNIC into binuclear (EPR-silent) complexes. Indeed, the oxidation of glutathione caused a shift in the equilibrium between these two forms of DNIC in the direction of diamagnetic binuclear DNIC (Figure 1).

However, at the HOCl : glutathione molar ratio of 2 : 1, there appeared an EPR signal characteristic of DNIC with phosphate ligands, which testified to the oxidation of thiolate ligands in binuclear DNIC (Figure 5(c)). The kinetics of this process is demonstrated in Figure 5(e). However, further increases in HOCl concentration resulted in the complete decomposition of DNIC (Figure 5(d)).

Obviously, the crucial role in the storage and transport of NO in biological systems is played by protein S-nitrosothiols and DNIC, which are at equilibrium with one another [19, 50, 51]. Previous studies established that after the injection of DNIC with low-molecular thiolate ligands into circulating blood their [Fe(NO)_2_] fragments bind predominantly to albumin [30]. At the same time, in blood plasma, albumin is the main target for hypohalous acids [52, 53].

**Figure 6:**
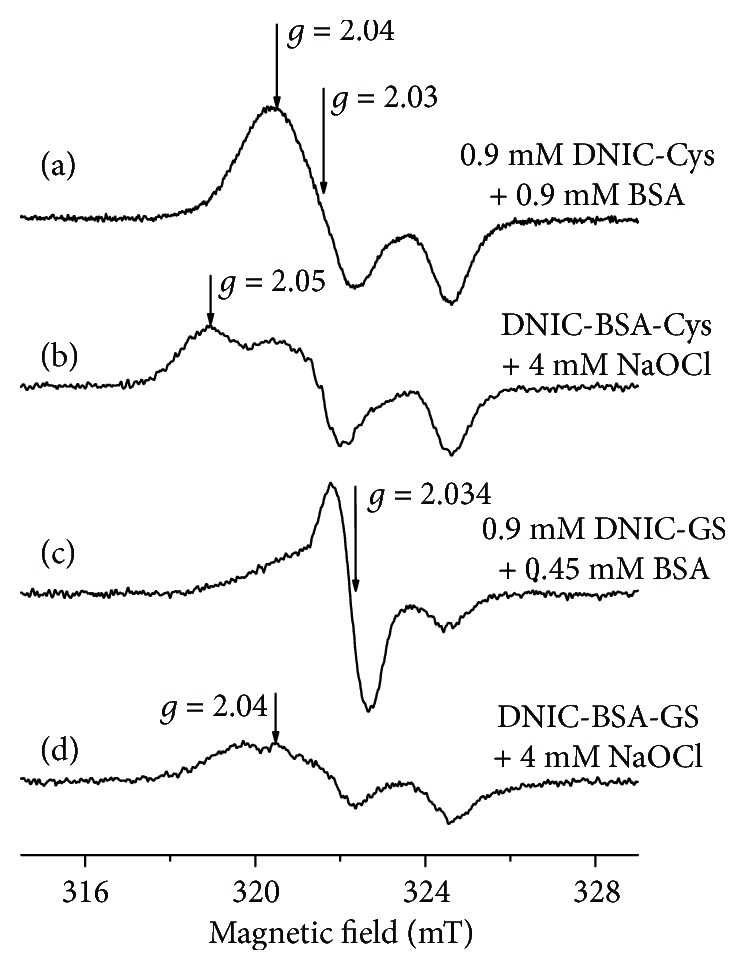
The EPR spectra of BSA-DNIC in the presence and in the absence of HOCl. Both types of albumin-bound DNIC (DNIC-BSA-Cys and DNIC-BSA-GS) were obtained by adding cysteine (DNIC-Cys) and glutathione (DNIC-GS) to bovine serum albumin (BSA), respectively. The EPR spectrum of DNIC-BSA-Cys (a). The EPR spectrum recorded after the addition of 4 mM HOCl to 0.9 mM DNIC-BSA-Cys (b). The EPR spectrum was recorded in the DNIC-GS+DNIC-BSA-GS mixture (c); the same after addition of 4 mM HOCl (d).

Thus, albumin-bound DNIC represent a convenient model for studying the interaction between protein-bound DNIC and HOCl.  Figure 6(a) shows the EPR spectra of DNIC with cysteine and the Cys34 residue of BSA as a ligand (DNIC-BSA-Cys) [29, 30]. The addition of HOCl to these DNIC initiated the appearance of a more asymmetric EPR signal (Figure 6(b)). Earlier, we found that similar changes in the shape of the EPR spectra take place during the oxidation of low-molecular thiolate ligands in albumin-bound DNIC and their substitution for the histidine residue of BSA [29]. However, the addition of excess DNIC with glutathione to BSA was accompanied by the formation of complexes containing albumin and glutathione (DNIC-BSA-GS) (Figure 6(c)).

In the given reaction system, protein-bound DNIC manifested higher resistance to HOCl than low-molecular DNIC with glutathione ligands (Figure 6(d)). Besides, these findings provide conclusive evidence that HOCl treatment of DNIC with low-molecular thiolate ligands initiates significant changes in their structure.

### 3.4. Comparison of HOCl Effects on Free and DNIC-Bound Glutathione

**Figure 7:**
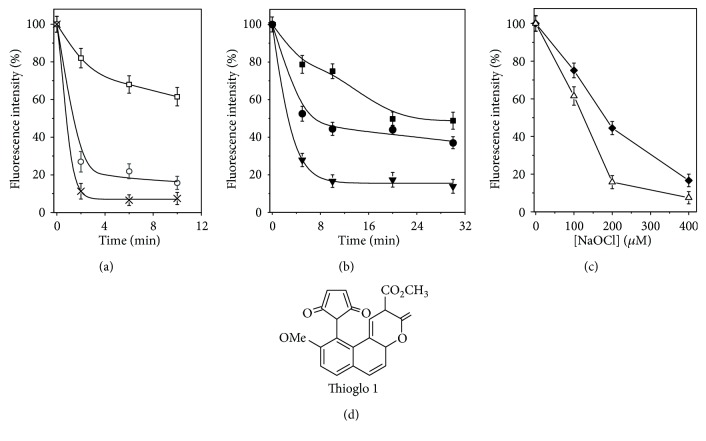
The reaction medium contained 100 *μ*M DNIC with glutathione (DNIC-GS) or 200 *μ*M GSH in PBS (pH 7.4) and different concentrations of HOCl. The incubation was carried out at ambient temperature (~25°C). The fluorescence spectra were recorded 3 min after the addition of ThioGlo 1 to the cell samples. The kinetics of oxidation of thiol groups of glutathione in the presence of 100 (**□**), 200 (**○**), and 400 *μ*M (**×**) HOCl (a). The kinetics of oxidation of thiol groups in DNIC glutathione ligands in the presence of 100 (■), 200 (●), and 400 *μ*M (**▼**) HOCl (b); the concentration of thiol groups in GSH (**∆**) and DNIC-GS (♦) after 10 min incubation with different concentrations of HOCl (c). The structural formula of ThioGlo 1 (d). The fluorescence of the samples prior to the addition of HOCl to the reaction medium was taken for 100%.

The effects of HOCl on thiol groups in GSH and glutathione ligands in DNIC were studied using ThioGlo 1 as a fluorescent probe (Figure 7(d)). The reaction of ThioGlo 1 with thiol groups gives a fluorescent product [36, 37]; its formation is inhibited after the preincubation of HOCl with both GSH and DNIC. However, the rate of HOCl-induced oxidation of glutathione ligands in DNIC was found to be much lower than in the case of free GSH (Figure 7). After 10 min incubation of equimolar concentrations of HOCl and glutathione, about 84% of thiol groups appeared to be oxidized (Figures 7(a) and 7(c)). A close level of oxidation of DNIC-bound glutathione was observed at the HOCl : glutathione molar ratio of 2 : 1 (Figures 7(b) and 7(c)). However, at the HOCl : DNIC-bound glutathione molar ratio of 1 : 1, only 55% of thiol groups lost their ability to interact with ThioGlo 1 (Figure 7(c)). These results suggest that glutathione binding to DNIC decreases the efficiency of the reaction of the thiol with HOCl: this reaction is less efficient upon the transformation of the mononuclear form of DNIC into the binuclear one.

## 4. Discussion

In our experiments, we studied the effect of hypochlorous acid on RBC, under conditions simulating halogenative stress. Activated neutrophils are able to generate *in vitro* up to 100 *μ*M HOCl [54, 55]. However, the local level of HOCl *in vivo* can be significantly higher. Indeed, the local concentration of HOCl in the inflammatory focus, calculated on the basis of the data given in [56], can reach 25-50 mM. Taking into account the potential for the formation of such high local concentrations of hypochlorous acid during a respiratory explosion, it can be assumed that even a large number of HOCl interceptors present in the blood plasma do not guarantee complete protection of RBC from HOCl-induced hemolysis.

Since an excess of hydrogen peroxide inhibits MPO, in order to minimize inactivation of the enzyme, usually no more than 100 *μ*M H_2_O_2_ is used [57]. This is the concentration we used in our experiments. It can be added that this concentration is quite comparable with the content of H_2_O_2_*in vivo* [58]. The concentration of DNIC used in our experiments with lysis of RBC (0.5-50 *μ*M) is also comparable to that found in biological systems [26, 59].

The totality of experimental data suggest that the inhibition of RBC lysis by DNIC with glutathione under conditions of oxidative/halogenative stress is conditioned by antiradical and antioxidant properties of DNIC. Indeed, similar to ascorbate and Trolox, vitamin E, taurine, flavonoids exert pronounced antioxidant effect by virtue of their ability to prevent RBC lysis in oxidative stress [12, 13, 16, 60–62]. The mechanism of this effect consists in inhibition of lipid peroxidation and protection of SH-groups of RBC proteins from oxidation.

It is known that nitrosyl complexes of heme and nonheme iron protect cells from oxidative stress by, e.g., inhibiting free radical oxidation induced by peroxides and superoxide [33, 43, 44, 63–65]. It was found also that the antioxidant effect is exerted either by DNIC proper or by NO and thiols that are at equilibrium with them [20, 43]. In addition, DNIC was found to inhibit lipid peroxidation in blood plasma and RBC membranes of animals with thermal traumas [31]. The binding of nitric oxide to DNIC inhibits the generation of peroxynitrite, one of the most potent oxidants known thus far [29, 30]. At the same time, the binding of Fe^2+^ ions within the composition of DNIC suppresses the generation of free radicals in Fenton and Haber-Weiss reactions [32, 57, 66]. Yet another interesting finding is that similar to H_2_O_2_, the Fenton reaction of HOCl with Fe^2+^ ions gives the hydroxyl radical (^·^OH) [67]:

(9) $$\begin{array}{c} {\text{Fe}}^{2+}+\text{HOCl}⟶{\text{Fe}}^{3+}{+}^{\cdot }\text{OH}+{\text{Cl}}^{-}. \end{array}$$

It should be noted that in our experiments, DNIC inhibited the lysis of RBC at concentrations comparable or substantially lower than those shown for other antioxidants (ascorbate, Trolox, and vitamin E) [16, 60, 61]. Moreover, ascorbate during hemolysis may exhibit prooxidant properties, reducing Fe^3+^ ions to Fe^2+^ [16]. It is also known that taurine in millimolar concentrations inhibited lysis of RBC and ROS production in them under oxidative stress induced by *tert*-butyl hydroperoxide [62].

We have also shown that a DEPMPO adduct with hydroxyl radical is formed in the reaction mixture containing HOCl and Fe^2+^ ions (Figure 4(f)). In its turn, the oxidation of GSH by the hydroxyl radical gives thiyl radicals of glutathione. The latter are generated upon decomposition of sulfenyl chloride induced by UV light or Fe^2+^ ions [38]. As it is known, sulfenyl and sulfonyl chlorides are formed as intermediate products in the course of thiol oxidation by HOCl [38]. The fact that in our study the generation of free radicals in the reaction mixture containing HOCl and DNIC with glutathione was absent (Figure 4(e)) led us to hypothesize that dinitrosyl iron complexes are not responsible either for ^·^OH generation (reaction (9)) or for decomposition of sulfenyl chloride.

Taking into account the relatively high value of the second-order rate constant for the HOCl reaction with reduced glutathione (*k*~1.24 × 10^8^ M^−1^ s^−1^) [68], we conjectured that cytoprotective and antioxidant activities of GSH observed in this series of our experiments can be attributed to the peculiarity of the aforementioned reaction. At the same time, our ThioGlo studies established that glutathione ligands in DNIC react with HOCl far less effectively than free GSH, presumably as a result of transfer of electron density in DNIC from glutathione sulfur atoms to iron and NO ligands [20].

It is important to note that NO ligands in DNIC are more resistant to HOCl than glutathione ligands. Indeed, after the oxidation of thiol components in DNIC by HOCl, their [Fe-NO_2_] fragments took part in the *de novo* synthesis of DNIC with phosphate ligands (Figure 6(a)). Most probably, the cytoprotective effect of DNIC with glutathione is not related to their interaction with HOCl, since in our studies it was manifested even at much lower (compared to HOCl) concentrations of DNIC. These conditions provoke the decomposition of DNIC with a concomitant release of NO able to suppress lipid peroxidation.

However, one should not rule out the fact that [Fe(NO)_2_] fragments in DNIC are endowed with the ability to react with free radical intermediates of lipid peroxidation. The reaction of nitrosyl complexes of iron or copper with molecular oxygen and H_2_O_2_, respectively, gives intermediates containing peroxynitrite (ОNOO^−^) bound to metal ions [69, 70]. It seems very probable that DNIC catalyze the formation of the organic peroxynitrite derivative ROONO and its further decomposition concomitant with the formation of nontoxic products (reactions (6)-(8)).

Human serum albumin manifests pronounced antioxidant activity [52]; however, after modification by hypohalous acids, this protein initiates enhanced generation of ROS by neutrophils [53]. At the same time, studies with albumin-bound DNIC established that the latter can hypothetically protect Cys and His residues from modification by HOCl. Other experiments showed that Cys and His residues are the main targets for HOCl in protein molecules [4, 71]. We succeeded in demonstrating that DNIC bound to the *β*-Cys93 residue in hemoglobin prevent the oxidative modification of hemoglobin by H_2_O_2_ [29, 30]. Under these conditions, the mechanism of the antioxidant effect of DNIC with glutathione might consist in the reduction of the oxoferryl form of heme (porphyrin-Fe(IV)=O) [43]. Indeed, reactions of hemoglobin with peroxides or HOCl give the ferryl form of hemoglobin possessing strong oxidizing activity and provoke numerous pathological conditions [72–74]. Thus, the oxoferryl form of heme causes autoxidation and binding of hemoglobin to the RBC membrane and induces lipid peroxidation [11, 74]. These interactions decrease the resistance of RBCs to hemolysis [54, 74, 75].

Studies by Vissers et al. demonstrated that HOCl-induced lysis of RBCs is associated with enhanced efflux of K^+^ from RBCs; it increases their resistance to deformation and decreases modification of RBC proteins [5, 6]. In addition, *tert*-butyl hydroperoxide and phospholipid hydroperoxides increase the permeability of RBC membranes and, as a consequence, enhance K^+^ leak from RBCs [76]. According to Diederich et al. [77], treatment of RBCs with *tert*-butyl hydroperoxide diminishes their deformation, while S-nitrosation of spectrins has no effect on their mechanical characteristics. Studies by other authors established that NO increases the deformability of RBCs, most probably as a result of the inhibition of K^+^-channels [78, 79] or the nitrosation of cytoskeletal and membrane proteins of the RBC [80–82]. Besides, the formation of S-nitrosothiols has a pronounced effect on the energy metabolism of RBCs [83, 84] and inhibits eryptosis [85]. It was hypothesized that NO is transferred from heme to the *β*-Cys93 residue of hemoglobin and then to SH-groups of the transmembrane anion-exchanger 1 protein (AE1) [8]. The finding that DNIC take part in selective nitrosation of protein SH-groups as NO^+^ donors [20, 22, 50, 86] led us to conclude that the ability of DNIC to protect RBCs from HOCl-induced lysis is determined by their antioxidant and regulatory properties.

## 5. Conclusions

Obtained data altogether testify to the crucial role of DNIC in protection of RBCs under conditions of oxidative/halogenative stress and in inflammation. In addition, DNIC with glutathione ligands are valuable tools for correcting a great number of pathological processes related to RBC lysis.

## Data Availability

The data used to support the findings of this study are included within the article. Additional information may be obtained from the corresponding author upon request.
